# High Resolution Spectral Domain Optical Coherence Tomography (SD-OCT) in Multiple Sclerosis: The First Follow Up Study over Two Years

**DOI:** 10.1371/journal.pone.0019843

**Published:** 2011-05-17

**Authors:** Nermin Serbecic, Fahmy Aboul-Enein, Sven C. Beutelspacher, Clemens Vass, Wolfgang Kristoferitsch, Hans Lassmann, Andreas Reitner, Ursula Schmidt-Erfurth

**Affiliations:** 1 Department of Ophthalmology, Medical University of Vienna, Vienna, Austria; 2 Department of Neurology, SMZ-Ost Donauspital, Vienna, Austria; 3 Department of Ophthalmology, Faculty of Medicine Mannheim, University of Heidelberg, Mannheim, Germany; 4 Brain Research Centre, Medical University of Vienna, Austria; Julius-Maximilians-Universität Würzburg, Germany

## Abstract

**Background:**

“Non-invasive, faster and less expensive than MRI” and “the eye is a window to the brain” are recent slogans promoting optical coherence tomography (OCT) as a new surrogate marker in multiple sclerosis (MS). Indeed, OCT allows for the first time a non-invasive visualization of axons of the central nervous system (CNS). Reduction of retina nerve fibre layer (RNFL) thickness was suggested to correlate with disease activity and duration. However, several issues are unclear: Do a few million axons, which build up both optic nerves, really resemble billions of CNS neurons? Does global CNS damage really result in global RNFL reduction? And if so, does global RNFL reduction really exist in all MS patients, and follow a slowly but steadily ongoing pattern? How can these (hypothesized) subtle global RNFL changes be reliably measured and separated from the rather gross RNFL changes caused by optic neuritis? Before generally being accepted, this interpretation needs further critical and objective validation.

**Methodology:**

We prospectively studied 37 MS patients with relapsing remitting (n = 27) and secondary progressive (n = 10) course on two occasions with a median interval of 22.4±0.5 months [range 19–27]. We used the high resolution spectral domain (SD-)OCT with the Spectralis 3.5 mm circle scan protocol with locked reference images and eye tracking mode. Patients with an attack of optic neuritis within 12 months prior to the onset of the study were excluded.

**Principal Findings:**

Although the disease was highly active over the observation period in more than half of the included relapsing remitting MS patients (19 patients/32 relapses) and the initial RNFL pattern showed a broad range, from normal to markedly reduced thickness, no significant changes between baseline and follow-up examinations could be detected.

**Conclusions:**

These results show that caution is required when using OCT for monitoring disease activity and global axonal injury in MS.

## Introduction

Multiple sclerosis (MS) is supposed to be a chronic inflammatory disease of the central nervous system (CNS) causing demyelination and axonal degeneration [1]. The cause of MS is still unknown. Underlying pathogenetic mechanisms are regarded to be complex and heterogeneous. Initially they may be triggered from outside of the CNS, but finally may become compartmentalized within the CNS [2]–[9]. In most patients the disease follows a relapsing-remitting (RRMS), and only rarely a progressive course right from the disease onset (PPMS, primary progressive MS). After an uncertain period of time, many RRMS patients change into secondary progression with or without superimposed relapses (SPMS, secondary progressive MS). Each MS patient follows his own individual disease course [10]. Neither the frequency and severity of relapses nor the disability in the first years after disease onset nor the lesion load in magnetic resonance images (MRI) nor any other established parameter nor biomarkers allow a reliable predication of individual disease courses so far [11]–[17]. Merely axonal injury is generally accepted to be the major pathological correlate of permanent disability in MS [18]. Thus, a method to measure reliably CNS axonal injury, could serve for disease monitoring in MS [19]–[20].

With OCT the retina can easily be visualized, its thickness measured and monitored over time. Initial findings supported OCT as a non-invasive and useful method. Baseline characteristics of the retinal nerve fibre layer (RNFL) of MS patients were found to be heterogeneous and ranged from normal to markedly reduced thickness [21]–[22]. The reduction of the RNFL seemed to correlate with the numbers of prior bouts of optic neuritis (ON), disease duration, and to the MS subtype - with most significant RNFL reduction in PPMS and SPMS (with and without ON) [23]–[24]. However, focal RNFL changes caused by ON are rather gross [25]–[27]. They must be clearly separated from any other global RNFL reduction, which is supposedly subtle, and may even lie under the detection limit of even advanced SD-OCT techniques [20], [26]–[27]. Long term studies performed with high resolution SD-OCT and rather large patient cohorts must provide the RNFL changes traceable for each included MS patient, to allow the readers a detailed interpretation [10], [28]. RNFL reduction rates per year, which are calculated by one (baseline) measurement only, can never replace carefully performed long term follow-up studies, or serve as ‘scientific proof’ for continuously ongoing RNFL reduction (‘RNFL thinning’) [10], [27]. In contrast, it remains yet completely unclear whether global RNFL reduction occurs continuously (‘RNFL thinning’) or stepwise, by accumulation of focal lesions, or both. Information about ‘RNFL reduction rates per year’ or ‘RNFL thinning’ should therefore be used with caution as they might be misleading. But whatever global RNFL reduction may be caused by, the most important issue is whether global RNFL reduction exists in all MS patients, or not and if so, whether subtle global RNFL changes can also be measured in short observation periods from 6 months to 2 years [23]–[24]. It is undisputed that advanced high resolution SD-OCT technique is a prerequisite [19], [20], [24], [26]–[29].

For this purpose we studied a large cohort of 27 RRMS and 10 SPMS patients with a new advanced SD-OCT technique over a long observation period of approximately 22.4±0.5 years. Moreover, despite partly intense disease activity with high relapse rates, none of the included patients had an ON within 12 months prior to onset of the study. In this study we analysed RNFL changes in relation to clinical data including age, gender, MS subtype, disease onset, disease duration, numbers of ON and relapses (prior to study entry, and during the observation period) as well as the treatment of each participant.

## Methods

### Participants

The study was approved by the local Ethics Committee (Commission of Medical Ethics of Vienna; Ethic Approval/Registration Number: EK-08-028-0308 and Ethical commission of the Medical University of Vienna; Ethic Approval/Registration Number: 414/2008). Informed written consent was obtained from all patients and volunteers before study entry.

We described the RNFL baseline characteristics of 59 MS (42 RRMS, 17 SPMS) patients in detail previously [22]. All 59 MS patients were invited to participate in our study. Thirty-seven MS (27 RRMS, 10 SPMS) patients consented to, whereas 22 patients had withdrawn from further participation. One of these patients had an embolic stroke of the left anterior cerebral artery, and one deteriorated rapidly (EDSS 8.0). The remaining 20 patients declared that they were not willing for further study participation (showing no other causes). However, they remained clinically stable (data not shown).

We studied the 37 MS patients (27 RRMS, 10 SPMS) on two occasions with a median interval of 22.4±0.5 months [range 19 to 27 months] prospectively. The patients were consecutively recruited. MS diagnosis was based on clinical course, MRI and on cerebrospinal fluid (CSF) analysis [30]. Other differential diagnoses were consequently ruled out [31]–[34]. Oligoclonal bands were found in the CSF samples of all MS-patients. Patients with other diseases that reduce RNFL thickness such as glaucoma, anterior ischemic optic neuropathy, high myopia, and congenital abnormalities of the optic nerves were excluded.

Baseline clinical neurological examinations, visual evoked potentials (VEP), and ophthalmologic examinations were performed within 7 days. No included patient had an ON within 12 months prior to the onset of the study. A summary of detailed demographic and clinical data for each included MS patient is given in table 1.

**Table 1:** Summary of demographic and clinical data.

				before 1^st^ OCT examination					follow-up		
	MS		age at	therapy	relapses*	ON		age at	age at	relapses*	therapy
No	subtype	sex	onset			right	left	baseline	follow-up		
**1**	RRMS	f	34.5	MITOX, GLAT, IFN(b), IFN(a)	7	0	0	40.5	42.25	3	natalizumab
**2**	RRMS	f	18.5	IFN(a), IFN(b)	4	0	0	23.5	25.25	3	natalizumab
**3**	RRMS	f	36.0	MITOX^1^, IFN(a)	7	0	0	42.0	43.5	2	natalizumab
**4**	RRMS	f	31.5	none	3	0	0	38.0	40.25	0	none
**5**	RRMS	m	40.0	IFN(a)	3	0	0	45.5	47.5	0	IFN(a)^2^; none
**6**	RRMS	f	28.5	IFN(b)	3	0	0	39.0	40.75	2	IFN(b), natalizumab^3^
**7**	RRMS	f	43.0	GLAT, IFN(b), none^4^	4	0	0	48.0	49.75	3	none, natalizumab
**8**	RRMS	f	40.0	none	2	0	0	42.25	44.0	0	none
**9**	RRMS	m	24.0	none	2	0	0	25.0	26.5	0	none
**10**	RRMS	f	18.0	GLAT, none^5^	2	0	0	19.75	21.5	1	none
**11**	RRMS	f	29.75	IFN(a)^6^, none	4	0	0	36.0	37.5	1	none
**12**	RRMS	m	31.0	IFN(b)	2	0	0	33.25	35.0	1	IFN(b)
**13**	RRMS	m	51.0	IFN(b)	2	0	0	52.0	54.75	0	IFN(b)
**14**	RRMS	f	23.75	none	2	0	0	27.0	28.5	0	none^7^
**15**	RRMS	m	27.5	GLAT	4	0	0	39.0	40.75	0	GLAT
**16**	RRMS	f	30.0	IFN(b)^8^, none	4	0	0	46.0	48.5	0	none
**17**	RRMS	m	39.0	IFN(c)	4	0	0	45.0	47.25	1	IFN(c)
**18**	RRMS	f	20.0	IFN(a)^9^, none	2	0	0	23.0	25.25	2	none
**19**	RRMS	f	16.0	GLAT	4	0	0	61.0	63.25	0	GLAT
**20**	RRMS	f	20.5	IFN(b)^10^, none	5	0	0	28.0	30.25	1	none
**21**	RRMS	f	26.0	IFN(a), IFN(b), MITOX^11^, none	9	1	1	32.0	34.0	0	none
**22**	RRMS	f	17.75	IFN(a), IFN(b)	6	1	3	19.75	21.75	1	IFN(b)^12^, natalizumab
**23**	RRMS	f	31.0	IFN(a), IFN(b)	4	1	0	36.0	38.25	0	IFN(b)
**24**	RRMS	f	20.0	IFN(b)	8	1	1	47.5	49.0	1	IFN(b)
**25**	RRMS	m	22.5	GLAT, IFN(a), IFN(b), natalizumab	10	0	1	42.5	44.0	0	natalizumab
**26**	RRMS	f	20.0	IFN(a)	3	0	4	41.0	42.5	0	IFN(a)
**27**	RRMS	m	31.0	GLAT^13^, none	2	0	1	33.0	35.25	0	none
**28**	SPMS	m	40.0	GLAT, MITOX^14^, none	3	0	0	46.5	48.5	0	none
**29**	SPMS	f	13.0	IFN(b), MITOX^15^, none	5	0	0	27.0	29.25	2	none
**30**	SPMS	f	38.0	GLAT, none	3	0	0	45.0	47.25	0	none
**31**	SPMS	f	33.5	none	3	0	0	56.0	58.25	0	none
**32**	SPMS	m	28.0	IFN(a), IFN(b)	11	0	0	47.25	49.0	1	IFN(b)
**33**	SPMS	m	25.0	IFN(c), GLAT, IFN(a), IFN(b)	10	1	1	47.5	49.75	1	IFN(b)
**34**	SPMS	m	22.0	IFN(b)	5	1	0	30.5	32.25	2	IFN(b)^16^, none
**35**	SPMS	f	16.0	IFN(a), MITOX^17^, none	6	0	2	44.25	46.5	2	none
**36**	SPMS	f	50.0	GLAT	4	0	2	53.5	55.75	2	GLAT
**37**	SPMS	m	29.0	IFN(b)	3	0	1	52.0	53.75	0	IFN(b)

**ON, optic neuritis**;

*, relapses treated with high dose steroid pulse therapy; no included patient had an ON within 12 months prior to the beginning of the study; **GLAT**, glatiramer-acetate 20 mg subcutaneous once daily; **MITOX**, mitoxantrone; **IFN(a)**, interferon beta 1a intramuscularly once per week; **IFN(b)**, interferon beta 1a (44 µg) subcutaneous trice per week; **IFN(c)**, interferon beta 1b (250 µg) subcutaneous alternate day;

1 , discontinued (48 mg mitoxantrone per m^2^ body surface); **none**, neither specific immunomodulatory or immunsuppressive therapy, drug holiday;

2 , drug withdrawal 9 months after 1^st^ OCT examination;

3 , start 7 months before 2^nd^ OCT examination;

4 , drug withdrawal 12 months before 1^st^ OCT examination;

5 , drug withdrawal 6 months before 1^st^ OCT examination;

6 , drug withdrawal 20 months before 1^st^ OCT examination;

7 , strict vegan diet caused vitamin B12 and folate deficiency;

8 , high titres of anti-interferon autoantibodies, drug withdrawal 14 months before 1^st^ OCT examination;

9 , drug withdrawal 25 months before 1^st^ OCT examination;

10 , drug withdrawal 8 months before 1^st^ OCT examination;

11 , mitoxantrone cumulative dose 96 mg per m^2^ body surface, drug withdrawal 10 months before 1^st^ OCT examination;

12 , change to natalizumab 2 months after 1^st^ OCT examination;

13 , drug withdrawal 6 months before 1^st^ OCT examination;

14 , mitoxantrone cumulative dose 92 mg per m^2^ body surface, drug withdrawal 10 months before 1^st^ OCT examination;

15 , mitoxantrone cumulative dose 92 mg per m^2^ body surface, drug withdrawal 26 months before 1^st^ OCT examination;

16 , drug withdrawal 5 months after 1^st^ OCT examination.

17 , mitoxantrone cumulative dose 108 mg per m^2^ body surface, drug withdrawal 27 months before 1^st^ OCT examination;

### High Resolution spectral domain OCT

The RNFL measurement was described in detail previously [22], [25]–[26]. Briefly, we used a high resolution SD-OCT which combines OCT technology with a confocal scanning laser ophthalmoscope (Heidelberg Engineering, Heidelberg, Germany, Spectralis software version 4.0.3.0, Eye Explorer Software 1.6.1.0). A special eye tracking mode (TrueTrac™) and high scanning speed allows the reduction of artefacts due to eye movement. Each peripapillary OCT is registered and locked to a reference image. OCT software can identify previous scan locations and “guide” the OCT laser beam to scan the same location again. To optimize the signal to noise ratio and image quality 16 frames (B scans) of the same scanning position were averaged with the Automatic Real-Time averaging mode (ART mode). All RNFL scans were acquired in the high-resolution acquisition mode allowing a more detailed differentiation of retinal layers, with pupil dilation. All RNFL scans were performed several times by one skilled and trained observer (N.S.) within one session until at least 3 high-quality scans were achieved and used for further analysis. The observer had no knowledge of any clinical data or the specific baseline data.

### Visual function testing, Visual Field Analysis, Visual evoked potentials (VEP)

Visual function testing, Visual Field Analysis and Visual evoked potentials were described in detail previously [22], [25].

### Statistics

The statistics used was described in detail previously [22].

## Results

**Figure 1:**
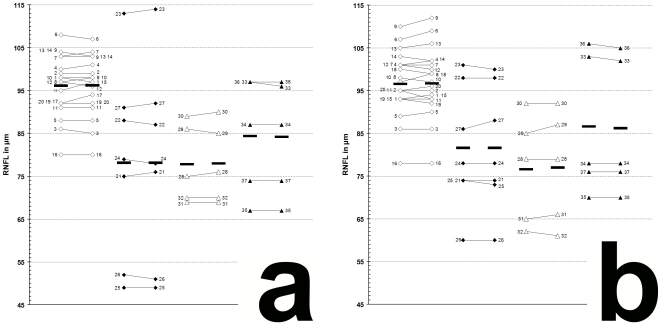
Global RNFL changes between baseline and follow-up examination. **a**, left eye; **b**, right eye; **1–37**, patient 1–37 (see table 1 for demographic and clinical data); **white squares**, RRMS without ON (baseline|—|follow-up); **black squares**, RRMS with ON (baseline|—|follow-up); **white triangles**, SPMS without ON (baseline|—|follow-up); **black triangles**, SPMS with ON (baseline|—|follow-up); **black bars**, means.

**Figure 2:**
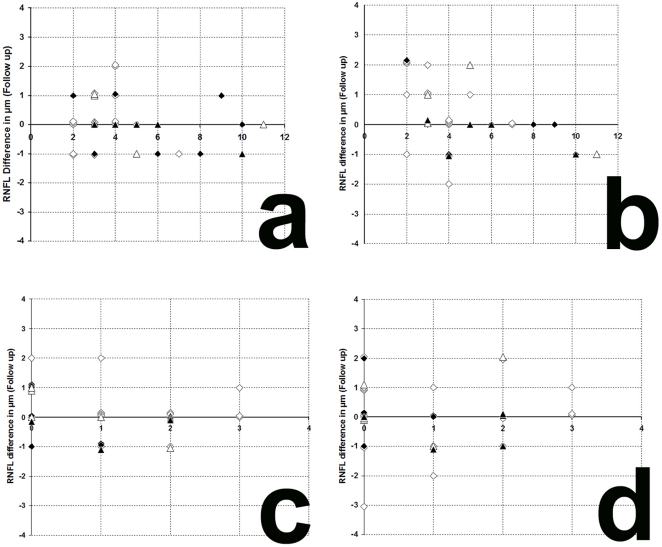
Variation of RNFL measurements. **a, b**: variation of RNFL measurements between baseline and follow-up examination in relation to the total relapses (without ON) before study entry. **c, d**: variation of RNFL measurements between baseline and follow-up examination in relation to the relapses (without ON) during the observation period. **a**, left eye; **b**, right eye; **c**, left eye; **d**, right eye; **white squares**, RRMS without ON; **black squares**, RRMS with ON; **white triangles**, SPMS without ON; **black triangles**, SPMS with ON.

The results of our study performed with the high resolution SD-OCT can be summarized briefly as follows. Over a median observation period of 22.4±0.5 months (range from 19 to 27 months) the RNFL measurements of each MS patient were unchanged compared to baseline (figure 1). The minimal changes (±2 µm) in some follow up scans were within the intersession variation (figure 1 and 2) [26], [27], [29]. Mean globally RNFL values were found highest in RRMS patients without ON. Mean RNFL were found lower in RRMS with ON and in SPMS with and without ON. However, in all 4 groups the individual RNFL values ranged from normal to markedly reduced, globally or at least in one of the six peripapillary sectors (figure 1) [22].

The visual and contrast acuity and sensitivity tests (ETDRS, Sloan and Pelli-Robson-charts), the colour vision test (Lanthony D-15) and the Humphrey visual field analysis and the visual evoked potentials showed also no changes compared to the baseline [22]. Again, neither age nor disease duration nor MS subtype was found correlated to RNFL changes.

## Discussion

In the past few years many OCT studies of MS patients, meta-analyses and reviews aroused great interest [19], [20]–[24]. OCT was suggested to be useful for monitoring the effects of neuroprotective therapies on MS disease course [19]–[20]. However, many core issues are still under intense discussion. Of most importance is that RNFL measurements performed under essentially the same conditions but by different OCT devices, differ significantly, and thus cannot be used or compared interchangeably [29]. The differences may be explained by the different technologies used (time domain vs. spectral domain), retinal segmentation algorithms, scanning speed, pupil dilation and other special technical features such as special eye tracking mode or high resolution acquisition modes or simply by intersession or inter-observer variability [29]. In contrast to rather gross ‘focal’ RNFL reduction rates about 5 µm to 40 µm, a global RNFL reduction about 2 µm to 4 µm per year (‘RNFL thinning’) was suggested in MS patients without ON [20], [23]. But ‘RNFL reduction rates per year’ or ‘RNFL thinning’ are statistical definitions only, and at least completely invalid, when not determined carefully in long-term follow up studies. Firstly, it is well known that the range of RNFL thickness in healthy individuals is wide (mean, 97.2 µm±9.7; range from 75 µm to 125 µm) and that RNFL thickness decreases with age [27]. Even when thousands of values for RNFL thickness would be averaged to a statistically highly significant and irrevocably mean, the initial RNFL thickness of the respective individual MS patient is unknown. Hence, a simple mathematical correlation between only one (baseline) RNFL value and disease duration cannot replace follow-up RNFL measurements during long observation periods. Recently, a minimum observation period of at least 2 years was suggested to be required to detect any subtle global RNFL changes in MS patients without ON, if they exist at all [20].

Secondly, it is yet unknown whether global RNFL reduction (apart from RNFL changes due to ON) really exists in all MS patients, and if so, when RNFL reduction may occur and whether it arises from continuously ongoing or relapsing (remitting) axonal changes. The hypothesized global RNFL reduction may already occur years before MS onset, or in very early stages of the disease, but also in later stages of the disease. Progressive axonal changes may be elusive in short but obvious in long observations periods. Accordingly, someone might assume, however that the hypothesized continuous but subtle RNFL reduction should occur when the disease course changes from the relapsing-remitting to the secondary progressive phase, or follows a progressive course right from the beginning in some cases of progressive relapsing-remitting MS. However, Henderson et al. reported that they did not detect any significant RNFL reduction (‘RNFL thinning’) in 34 MS patients with progressive disease (18 PPMS, 16 SPMS) in a rather long observation period (median 575 days, range from 411 to 895) [24]. From that it is conceivable that (hypothesized) global RNFL changes either do not exist, or that global RNFL changes are too subtle to be detected with conventional TD-OCT or that the observation period was too short. However, most clinical trials are frequently around 6 to 12 months. Anyway, the supposed RNFL reduction rates about 2 µm per year are below the detection limits of time domain- and even most SD-OCT devices (variations up to 5 µm) [26], [29]. Thirdly, rather gross and focal RNFL changes can hardly be separated from any other hypothesized subtle, progressive RNFL reduction, and thus may interfere with any predication of subtle global RNFL changes.

Finally, whatever, the underlying pathogenetic mechanisms might be, it is completely unclear whether the damage of few axons along the optic nerves represent really the anatomic and functional fate of hundred of billions neurons in the entire CNS. In fact, only rarely the MRI lesion load correlates to the clinical course of MS patients. Consequently, the question arises why the RNFL thickness should indeed correlate strictly to MRI parameters and the clinical course of MS patients. In other words, why should an hypothesized retrograde trans-synaptic axonal degeneration have such an effect on the RNFL, but in contrast does an ON not lead to widespread anterograde axonal degeneration in the whole CNS? To the best of our knowledge ON was never found to be correlated to, or proclaimed to cause diffuse axonal degeneration in the whole CNS, respectively brain atrophy.

In conclusion, even if most of the MS patients follow a primary relapsing-remitting course with clinical stable intervals over months, years or sometimes even decades, that does of course not rule out subclinical ‘silent’ ongoing degenerative changes [35]. Whether such hypothesized changes really exist in all MS patients remain to be critically and objectively evaluated. As others we propose standardized technical requirements [24], [27], [29], [34]. However, in our long term study performed with advanced SD-OCT technique we did not find RNFL reduction (‘RNFL thinning’) in a well defined cohort of RRMS and SPMS with partly intense disease activity. Our data suggest that OCT can yet not replace MRI, or even serve as new surrogate marker in MS.
